# CMMS-GCL: cross-modality metabolic stability prediction with graph contrastive learning

**DOI:** 10.1093/bioinformatics/btad503

**Published:** 2023-08-12

**Authors:** Bing-Xue Du, Yahui Long, Xiaoli Li, Min Wu, Jian-Yu Shi

**Affiliations:** School of Life Sciences, Northwestern Polytechnical University, Xi’an 710072, China; Institute for Infocomm Research (I^2^R), Agency for Science, Technology and Research (A*STAR), Singapore 138632, Singapore; Singapore Immunology Network (SIgN), Agency for Science, Technology and Research (A*STAR), Singapore 138648, Singapore; Institute for Infocomm Research (I^2^R), Agency for Science, Technology and Research (A*STAR), Singapore 138632, Singapore; Institute for Infocomm Research (I^2^R), Agency for Science, Technology and Research (A*STAR), Singapore 138632, Singapore; School of Life Sciences, Northwestern Polytechnical University, Xi’an 710072, China

## Abstract

**Motivation:**

Metabolic stability plays a crucial role in the early stages of drug discovery and development. Accurately modeling and predicting molecular metabolic stability has great potential for the efficient screening of drug candidates as well as the optimization of lead compounds. Considering wet-lab experiment is time-consuming, laborious, and expensive, *in silico* prediction of metabolic stability is an alternative choice. However, few computational methods have been developed to address this task. In addition, it remains a significant challenge to explain key functional groups determining metabolic stability.

**Results:**

To address these issues, we develop a novel cross-modality graph contrastive learning model named CMMS-GCL for predicting the metabolic stability of drug candidates. In our framework, we design deep learning methods to extract features for molecules from two modality data, i.e. SMILES sequence and molecule graph. In particular, for the sequence data, we design a multihead attention BiGRU-based encoder to preserve the context of symbols to learn sequence representations of molecules. For the graph data, we propose a graph contrastive learning-based encoder to learn structure representations by effectively capturing the consistencies between local and global structures. We further exploit fully connected neural networks to combine the sequence and structure representations for model training. Extensive experimental results on two datasets demonstrate that our CMMS-GCL consistently outperforms seven state-of-the-art methods. Furthermore, a collection of case studies on sequence data and statistical analyses of the graph structure module strengthens the validation of the interpretability of crucial functional groups recognized by CMMS-GCL. Overall, CMMS-GCL can serve as an effective and interpretable tool for predicting metabolic stability, identifying critical functional groups, and thus facilitating the drug discovery process and lead compound optimization.

**Availability and implementation:**

The code and data underlying this article are freely available at https://github.com/dubingxue/CMMS-GCL.

## 1 Introduction

Metabolic stability, which refers to the extent and rate of drug metabolism in the body, plays a crucial role in the early stages of drug discovery and development (Pritchard *et al.* 2003). It significantly impacts various pharmacokinetic (PK) processes, such as oral bioavailability, the volume of distribution, clearance, half-life, and toxicity, ultimately influencing the appropriate drug dose and frequency (Davies *et al.* 2020, Gajula *et al.* 2021). Therefore, improving the metabolic stability of hit/lead compounds in the early phases of drug discovery has a pivotal role w.r.t. drug candidate screening and lead compound optimization (Kirchmair *et al.* 2015).

Most of the early metabolic stability assays are conducted *in vitro* by incubating hit/lead compounds with liver microsomes (LMs) to assess their metabolic properties (Gajula *et al.* 2021). Notably, LMs are abundant in subcellular components containing cytochrome P450 (CYP450) enzymes, which play a crucial role in drug metabolism, and their assays provide preliminary guidance for more definitive PK assessments (Słoczyńska *et al.* 2019). Furthermore, *in vitro* mouse liver microsomes (MLMs), rat liver microsomes (RLMs), and human liver microsomes (HLMs) have emerged as essential approaches for initial assessment of metabolic stability (Mak *et al.* 2022, Rodríguez-Pérez *et al.* 2023). However, experimental determination of molecular metabolic stability is time-consuming, laborious, and expensive (Sodhi and Benet 2021). Therefore, there is an urgent need to develop computational methods to assist experimental screenings of metabolic stability.

In the past several years, traditional machine learning-based methods have been developed for metabolic stability prediction. For example, Perryman *et al.* (2016) first collected a dataset of MLM compounds from PubChem and presented a Bayesian model to predict molecular metabolic stability using nine molecular descriptors (e.g. fingerprints). Podlewska and Kafel (2018) built an online platform called MetStabOn for metabolic stability prediction, where multiple kinds of traditional machine learning methods were assembled, such as k-nearest neighbor (kNN), Naïve Bayes (NB), and random forest (RF). MetStabOn takes PaDEL-descriptors and extended fingerprints as input and can be applied for MLMs, RLMs, and HLMs data. Recently, Ryu *et al.* (2022) first generated an in-house HLMs dataset and introduced a RF model to evaluate metabolic stability with molecular descriptors as inputs. However, these methods take fingerprint or descriptor information into account only, while ignoring the molecule structural information, which results in suboptimal performance.

The rapid development of machine learning has led to the widespread application of graph neural networks (GNNs) in various biological research fields, such as drug–target interaction prediction (Long *et al.* 2022, Wang *et al.* 2022), molecular property prediction (Wu *et al.* 2018, Chen *et al.* 2021), and ADMET prediction (Feinberg *et al.* 2020, Xiong *et al.* 2021, Du *et al.* 2023). The compound molecule can be characterized as a graph structure, and motivated by this fact, several studies have recently attempted to use GNNs to predict metabolic stability. For example, Renn *et al.* (2021) first converted compound SMILES sequences to graph structures and then implemented graph convolutional networks on the graph structures for metabolic stability prediction. This method learned molecule representations by capturing both local and global chemical properties. Very recently, Rodríguez-Pérez *et al.* (2023) proposed a GNN-based assemble method called MT-GNN to evaluate metabolic stability, which can make predictions across multiple species. Besides, Li *et al.* (2022) developed a directed message-passing neural network (D-MPNN) for metabolic stability prediction based on the molecular graph.

Nevertheless, existing methods have two main limitations. First, most of them focus solely on using either SMILES sequence or molecular structure for metabolic stability prediction. While SMILES sequences provide information about the context of symbols and molecular structure determines the physical and chemical properties of molecules, combining both modalities allows combining SMILES sequence with molecular structure allows deep learning methods for learning more informative features than single modality sequence or structure data. Second, identifying the key functional groups that determine metabolic stability is crucial for lead compound optimization and drug design. Unfortunately, only a few previous methods have been developed to deal with this task.

To address these challenges, we propose a cross-modality graph contrastive learning model named CMMS-GCL for metabolic stability prediction. In the model, we design a dual representation of learning channels for molecules. Specifically, with SMILES sequence data as inputs, we first design a multihead Bi-directional Gated Recurrent Unit (BiGRU)-based encoder to learn the sequence representation of molecules by fully preserving the context of symbols. To capture the consistencies between local and global structures, we propose a graph contrastive learning framework to learn different structure representations. To the best of our knowledge, CMMS-GCL is the first method to simultaneously consider SMILES sequence and molecule structure for metabolic stability prediction. Comprehensive experiments show that our CMMS-GCL outperforms state-of-the-art methods. In addition, case studies further validate the interpretability of critical functional groups identified by CMMS-GCL. Overall, the main contributions can be summarized as follows:

- We propose a novel graph contrastive learning model named CMMS-GCL for metabolic stability prediction. Specifically, CMMS-GCL is an interpretable model, adept at identifying key functional groups crucial for metabolic stability. The model achieves this by analyzing specific sequence cases, with further validation provided by statistical analyses rooted in the graph structure.
- We design a new cross-modality representation learning framework where SMILES sequence and molecule structures are considered simultaneously to learn molecule features. To the best of our knowledge, this is the first attempt to integrate both modality data for effective metabolic stability prediction.
- Comprehensive experiments are conducted on two datasets and the results show that our CMMS-GCL outperforms 7 state-of-the-art methods. Case studies on several stable/unstable molecules further validate the interpretability of key functional groups identified by CMMS-GCL.

## 2 Methodology

### 2.1 Problem formulation

We model the metabolic stability prediction problem as a classification task. Assuming that we are given a set of *n* compounds $D=\left\{D_{i}|i\in \left\{1,\ldots ,n\right\}\right\}$ and a set of metabolic stability labels $y=\left\{y_{i}|y_{i}\in \{0,1\},i\in \left\{1,\ldots ,n\right\}\right\}$ for each compound $D_{i}$, where $y_{i}=1$ represents the *i*-th compound is stable; otherwise unstable. Our main task is to train a classifier $f_{\theta }$ to assign probability scores $\hat{y}$ to the compounds to determine whether these compounds are stable. Note that θ denotes the classifier parameters.

### 2.2 Overview of CMMS-GCL

**Figure 1.**
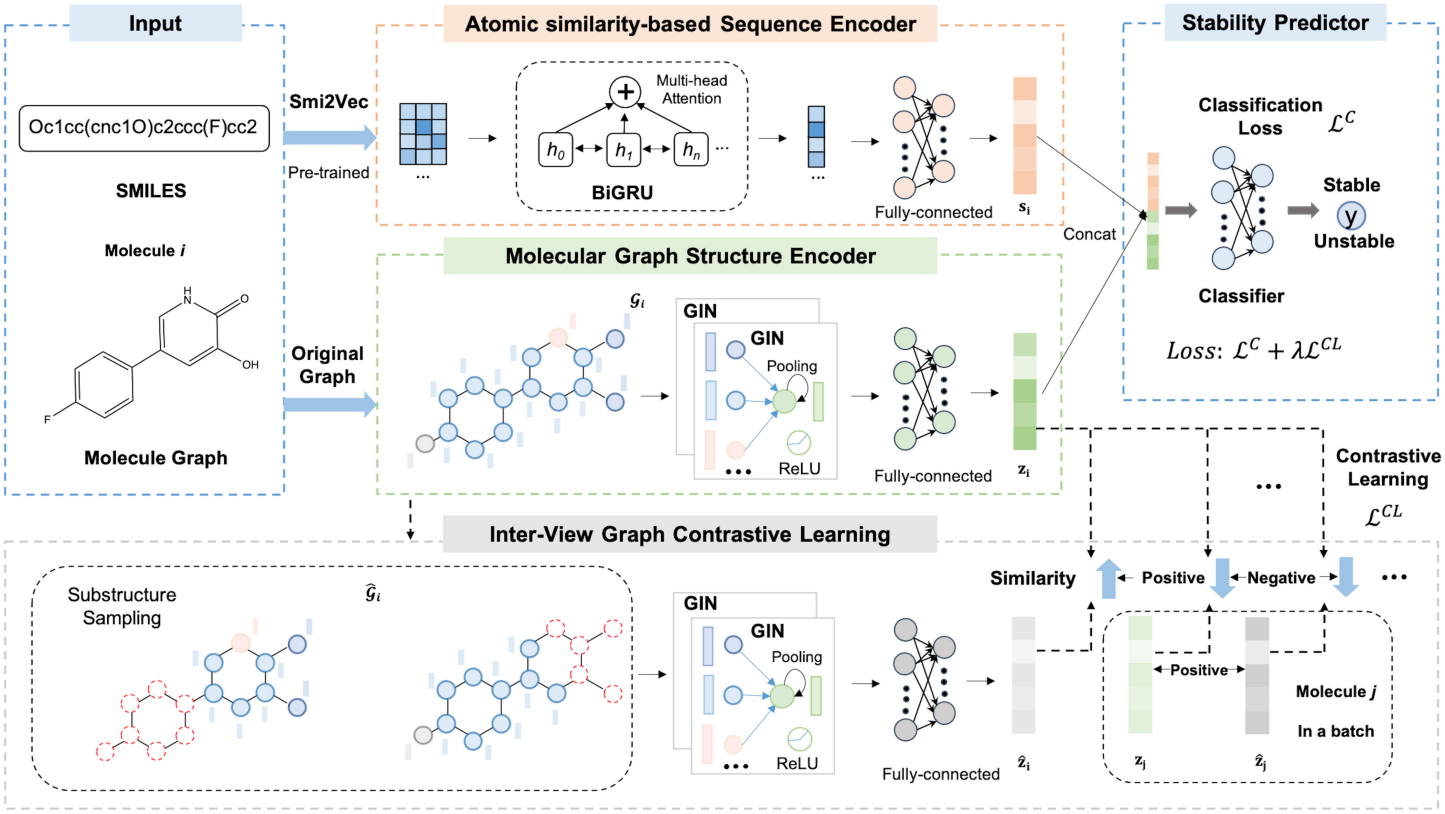
The overall framework of CMMS-GCL for metabolic stability prediction. CMMS-GCL is a cross-modality fusion framework augmented via graph contrastive learning, which contains an atomic similarity-based sequence encoder, a molecular graph structure encoder, an inter-view graph contrastive learning, and a stability predictor. Specifically, the atomic similarity-based sequence encoder uses a multihead BiGRU to generate molecular sequence embeddings, where the initial features are obtained by a pretrained Sim2Vec. The molecule graph structure encoder generates molecule embeddings by two-layer GINs. The inter-view graph contrastive learning module augments the original molecular graph by substructure sampling and then contrasts the augmented molecular graph embeddings and the original molecular graph embeddings. Finally, the sequence embeddings and graph embeddings are concatenated and fed into the final predictor for metabolic stability prediction.

In this article, we propose a novel cross-modality graph contrastive learning framework called CMMS-GCL for metabolic stability prediction. As shown in Fig. 1, CMMS-GCL mainly consists of an atomic similarity-based sequence encoder, a molecular graph structure encoder, an inter-view graph contrastive learning and a stability predictor. In particular, the atomic similarity-based sequence encoder captures the atomic semantically similar mapping, the molecular graph structure encoder represents the global structure of atoms and bonds, the interview graph contrastive learning module generates local structure and preserves consistencies with global structure, and the stability predictor estimates the stability of a given molecular structure based on the learned representations. Next, we will introduce each module in detail.

### 2.3 Atomic similarity-based sequence encoder

Compounds are usually represented by the SMILES (Simplified Molecular-Input Line-Entry System). The local chemical context of an atom reveals its functionality in the SMILES sequence. To capture the context information, we first learn the preliminary features of atoms from SMILES sequence data using Smi2Vec (Lin *et al.* 2020a,b), which is shown to be capable of preserving the proximity of semantically similar mappings in the embedding space. Following that, we further design an atomic similarity-based sequence encoder to learn representations of compounds. Specifically, the Smi2Vec algorithm first divides a given SMILES string into individual atom or symbol and then looks up the embedding for each atom from a pretrained embedding dictionary. If an atom is not in the dictionary, a random embedding is generated. With the output of Smi2Vec, we can derive the pretrained atom representations $Z\in R^{m\times d_{1}}$, where each row represents the pretrained representation of an atom. *m* and $d_{1}$ denote the number of atoms and the dimension of representations, respectively.

After deriving the preliminary atom representations, we further design a BiGRU-based encoder to learn representations for compounds. GRU aims to enforce every recurrent unit to preserve the dependencies of different time scales adaptively. Therefore, as a variant of GRU, BiGRU allows the encoder to capture the local chemical context of atoms. Mathematically, the hidden state $h_{t}$ of the *t*-th atom in the sequence can be formulated as follows: where $\vec{\text{GRU}}(\cdot )$ and $\vec{\text{GRU}}(\cdot )$ represent the forward and backward GRUs, respectively, which captures the interdependence between physically adjacent atoms within the sequence. $z_{t}$ is the preliminary representation vector of the *t*-th atom in the sequence. $\vec{z_{t-1}}$ and $\vec{z_{t+1}}$ denote the hidden states of the (t−1)-th and (t+1)-th atoms from the forward and backward GRUs, respectively. As the output of the BiGRU model, we can generate the updated atom representation $H\in R^{m\times d_{2}}$ with dimension $d_{2}$.


(1) $$h_{t}=\left[\vec{\text{GRU}}\left(z_{t},\vec{z_{t-1}}\right),\vec{\text{GRU}}\left(z_{t},\vec{z_{t+1}}\right)\right],$$

Previous study shows that key functional groups (i.e. molecule substructure) play a critical role in metabolic stability (Li *et al.* 2022). To effectively identify key functional groups, we introduce an attention mechanism (Zheng *et al.* 2020) to the BiGRU model. In particular, given a molecule *i*, we first learn the importance of all atoms by the following attention coefficient: where $\alpha_{ij}\in R^{1\times m}$ is attention coefficient that denotes the importance of atom *j* to the molecule *i*. MLP(⋅) is a trainable feedforward neural network with an activation function (i.e. tanh). Softmax[⋅] is a normalization function that ensures the sum of attention scores of all atoms is equal to 1. Subsequently, we derive the molecule representation $s_{i}$ by aggregating the representation of all the atoms according to their attention coefficients: where $FC_{s}(\cdot )$ is a fully connected neural network.


(2) $$\alpha_{ij}=\text{Softmax}\left[\text{MLP}\left(H\right)\right],$$

(3) $$s_{i}={\text{FC}}_{s}\left(\sum_{j=1}^{m}\alpha_{ij}h_{j}\right),$$

### 2.4 Molecular graph structure encoder

In this section, we introduce the graph isomorphism network (GIN) to learn the representation of molecules from molecular structures. Given a molecule *i*, let us denote its graph structure as G={V,E}, where V is a set of *N* nodes (i.e. atoms) and E is a set of edges (i.e. chemical bonds connecting atoms). We denote $A\in R^{N\times N}$ as the adjacency matrix of the graph, in which $a_{vu}=1$ indicates atoms *v* and *u* are connected by a bond while $a_{vu}=0$ indicates atoms *v* and *u* are disconnected by a bond. *N* is the number of atoms. The initial input features of the molecule *i* are defined by several atom-level properties such as the atom symbol, the number of adjacent atoms, the number of adjacent hydrogens, the implicit value of the atom, and the atom occurrence in an aromatic structure.

For each atom *v* in the molecular graph G, GIN learns its representation by iteratively aggregating the representations of its neighbors. Specifically, the representation $h_{v}^{\left(k\right)}$ for atom *v* in the *k*th layer is defined as follows: where $\epsilon^{\left(k\right)}$ is a learnable weight parameter. $h_{v}^{\left(k\right)}$ is the representation of node *v* at the *k*-th iteration. MLP(⋅) is a feedforward neural network. $h_{v}^{\left(k-1\right)}$ is the representation of node *v* at the (k−1)-th iteration. N(v) denotes the set of neighbors of the node *v*. With *K* iterations, the representation $h_{v}$ can effectively capture *K*-hop neighborhood information. Eventually, we take the output of the *K*-th iteration as the final representation $h_{v}$ for the atom *v*. To obtain the molecule representation $p_{i}\in R^{1\times d_{3}}$ with dimension $d_{3}$, we first implement global max-pooling and global mean-pooling on the atom representation $h_{v}$, respectively, and then concatenate two pooled representation vectors.


(4) $$h_{v}^{\left(k\right)}=\text{MLP}\left(\left(1+\epsilon^{\left(k\right)}\right)\cdot h_{v}^{\left(k-1\right)}+\sum_{u\in N(v)}h_{u}^{\left(k-1\right)}\right),k=0,1$$

(5) $$p_{i}=\mathrm{CONCAT}(\text{max}\left\{\;h_{v}^{i}(u)\right\},\text{mean}\left\{\;h_{v}^{i}(u)\right\}).$$

### 2.5 Inter-view graph contrastive learning

Inspired by the study that preserving consistencies between local and global structures in a graph is demonstrated to improve graph representation learning (You *et al.* 2020), we further introduce graph contrastive learning to enhance the representation learning for molecules.

Given a molecular graph G={V,E}, we adopt substructure sampling-based augmentation to generate a local subgraph, denoted as $\hat{G}=\{\hat{V},\hat{E}\}$, where $\hat{V}$ and $\hat{E}$ are the sets of nodes and edges respectively and $\left|\hat{V}|\leq k|V\right|$. Suppose we randomly sample a mini-batch of *Q* molecular graphs and, through data augmentation, derive an equal number of corresponding subgraphs to enhance the learning process. In particular, for a given molecule *i*, its graph representation and its corresponding graph representation from the augmented subgraph form a positive pair, while its representation and the representations of the remaining 2(*Q*-1) graphs/subgraphs form negative pairs. The primary idea of graph contrastive learning is to maximize the agreement between the representations of positive pairs while minimizing the agreement between the representations of negative pairs (Chen *et al.* 2020). With a given molecule graph *i* and its corresponding subgraph as inputs, we can attain its representations $z_{i}$ and its subgraph representation $\hat{z_{i}}$ via GIN-based encoder. The contrastive loss is defined as follows: where $\text{sim}(z_{i},\hat{z_{i}})=z_{i}^{⊤}\hat{z_{i}}/\|uz_{i}||||\hat{z_{i}}||$ denotes the similarity of the representations $z_{i}$ and $\hat{z_{i}}$, i.e. cosine similarity. τ is the temperature parameter (set to 0.2 by default). As such, for the input batch samples, their overall contrastive loss is defined as follows:


(6) $$\ell_{\text{contr}}^{z_{i},\hat{z_{i}}}=-\text{log}\;\frac{\;\text{exp}\;\left(\text{sim}\left(z_{i},\hat{z_{i}}\right)/\tau \right)}{\sum_{k=1,k\neq i}^{2Q}\;\text{exp}\;\left(\text{sim}\left(z_{i},z_{k}\right)/\tau \right)},$$

(7) $$L^{CL}=\frac{1}{2Q}\sum_{i=1}^{Q}\left(\ell_{\text{contr}}^{z_{i},\hat{z_{i}}}+\ell_{\text{contr}}^{\hat{z_{i}},z_{i}}\right).$$

### 2.6 Stability predictor

For a given molecule *i*, after deriving its sequence representation $s_{i}$ and structure representation $z_{i}$, we first concatenate them and then feed them into a classifier. where ${\hat{y}}_{i}$ is a probability score that represents the possibility of being stable or unstable. The classification loss function is defined as follows: where $y_{i}\in \{0,1\}$ is the true label (i.e. stable or unstable), and *Q* is the number of molecules in the mini-batch samples. Subsequently, the overall loss function L is defined in Equation (10). where λ is the weight coefficient that is used to trade-off the contributions of classification loss and contrastive learning loss.


(8) $${\hat{y}}_{i}=\text{FC}\left[\mathrm{CONCAT}(z_{i},s_{i})\right],$$

(9) $$L^{C}=-\sum_{i=1}^{Q}y_{i}\cdot \;\text{log}\;\sigma ({\hat{y}}_{i})+(1-y_{i})\cdot \;\text{log}\;\sigma (1-{\hat{y}}_{i}),$$

(10) $$L=L^{C}+\lambda L^{CL},$$

## 3 Experiments

In this section, we first introduce the experimental setups and then validate the performance of our proposed CMMS-GCL by comparing it with baselines, conducting an ablation study, and presenting a case study.

### 3.1 Experimental setups

#### 3.1.1 Datasets and evaluation metrics

The first metabolic stability dataset used in our experiments denoted as HLM dataset, was obtained from literature (Li *et al.* 2022). The authors collected human liver microsomal data from the ChEMBL bioactivity database. As defined in an existing study (Shah *et al.* 2020), molecules with a half-life of <30 min or 50% remaining after 30 min are considered stable; otherwise unstable. Hence, in this work, we determined the metabolic stability of molecules with a threshold of half-life within 30 min. We removed duplicate molecules and finally obtained 5878 molecules for HLM dataset, including 3784 stable and 2094 unstable compounds. In addition, following (Li *et al.* 2022), we evaluated the performance of our CMMS-GCL model on an external dataset consisting of a total of 111 molecules, of which 82 are stable and 29 are unstable. Table 1 summarizes the details of these two datasets.

**Table 1.** Statistics of metabolic stability datesets.

Dataset	Total	Positive (stable)	Negative (unstable)
HLM dataset	5878	3784	2094
External dataset	111	82	29

**Figure 2.**
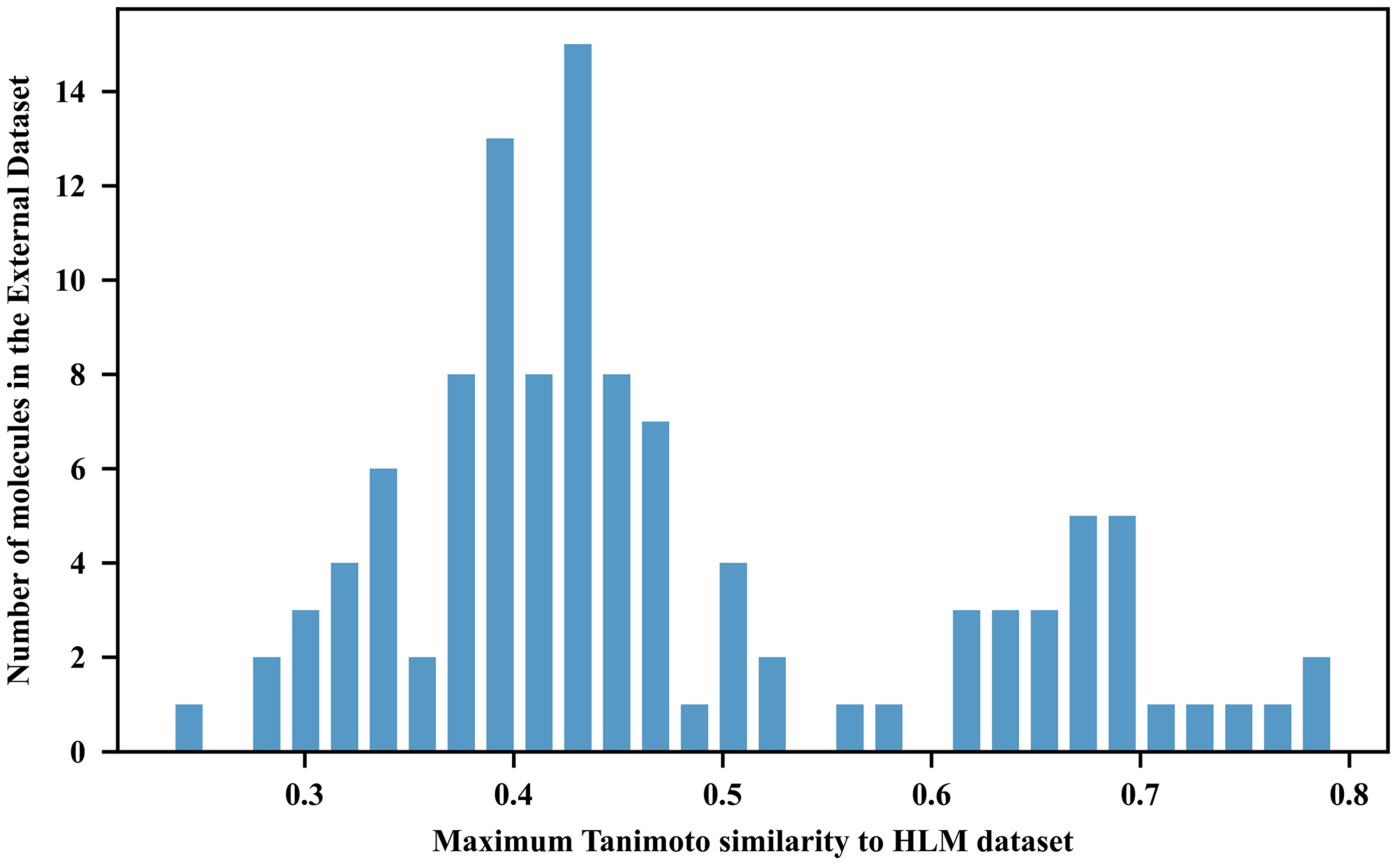
Distribution of the maximum Tanimoto similarity scores of each molecule in the external dataset when compared with the entire HLM dataset.

Furthermore, we assessed the molecular structural similarity between the molecules in the External and HLM datasets. We used the Extended Connectivity Fingerprints (ECFP) for molecules and calculated their Tanimoto similarity, a robust measure of structural similarity between molecules (Bajusz *et al.* 2015, Ucak *et al.* 2021). Note that the range of the Tanimoto similarity is between 0 and 1, where 0 indicates entirely dissimilar structures and 1 means identical molecules. Given a molecule in the external dataset, we calculated its Tanimoto similarity scores to all the molecules in the HLM dataset, and considered the maximum similarity score as the similarity between this given molecule and the HLM dataset (Fig. 2). Following the previous study (He *et al.* 2022), compounds are generally classified into three categories based on their Tanimoto similarity scores: up to 0.5 (dissimilar), between 0.5 and 0.7 (moderately similar), and greater than 0.7 (highly similar). Within these similarity scores in our dataset, we found 70.27% were dissimilar, 23.42% were moderately similar, and a minor 6.31% were highly similar, resulting in an overall mean Tanimoto similarity score of 0.47, thus underscoring the low degree of similarity between our external and HLM datasets.

We performed two types of evaluation experiments on the above two datasets. In particular, we implemented 10-fold cross-validation on the HLM dataset for performance evaluation. And we repeat the experiment 10 times and take average values as the final result on the external dataset. We further conducted the external independent evaluation, by training the model on the HLM dataset and testing on the external dataset. Three well-known performance metrics are utilized for evaluation, including the average area under the receiver operating characteristic curve (AUC), Accuracy, F1 score, and Matthew’s correlation coefficient (MCC). For all these three metrics, a higher value means better performance.

#### 3.1.2 Implementation details

In the atomic similarity-based sequence encoder, the dimensions $d_{1}$ and $d_{2}$ of the pretrained and output atom representations are set to 100 and 200, respectively. In the molecular graph structure encoder, we initially represented each node of the input molecule graph using a binary atom feature vector with 84 dimensions. We set the number *K* of GIN layers as 2. In the stability predictor, the dimensions of the input layer, hidden layers, and output layer are set to 768, 256, and 1, respectively.

To train the model of CMMS-GCL, we set training epochs and learning rate as 200 and 0.0005, respectively. Adam (Kingma and Ba. 2015) is selected as the optimizer. For data augmentation, the sampling ratio *k* is set as 0.4. We evaluate the influence of the weight coefficient λ by testing values in {0.01,0.05,0.1,0.25,0.5,0.75,1,1.5,2} and find that the best performance is achieved with λ = 0.1.

Furthermore, CMMS-GCL is implemented in Python 3.8 and PyTorch 2.0, along with functions from PyG 2.3.1, Networkx 2.8.4, Scikit-learn 1.2.2, Numpy 1.23.5, Pandas 2.0.2, and RDKit 2023.03.1.

### 3.2 Comparisons with baselines

To evaluate the performance of the proposed method, we compare CMMS-GCL with seven state-of-the-art methods, including three traditional machine learning-based methods and four deep learning-based methods, summarized as follows:

- FP-GBDT (Li *et al.* 2022): we implemented GBDT in the exact same way as that in (Li *et al.* 2022), which used ECFP fingerprints as input with the optimized parameters setting where the maximum depth of the tree is 4, the number of decision trees is 1200 and the minimum number of samples required to be at a leaf node was set to 9.
- FP-XGBoost (Li *et al.* 2022): the implementation of XGBoost is similar to FP-GBDT, which also used ECFP fingerprints as input. Optimized parameter settings adopted by the references include a maximum tree depth of 3, an ensemble of 900 decision trees, and a minimum requirement of 5 samples per leaf node.
- PredMS (Ryu *et al.* 2022): it used highly important molecular descriptors features as input and then sent into RF-based feature selection with the parameter setting where the maximum depth of the tree is 30, the number of decision trees is 500 and the minimum number of samples required to be at a leaf node was set to 3.
- GCN (Renn *et al.* 2021): it implemented the same molecule graph features of CMMS-GCL and then used a two-layer GCN to a feed-forward neural network. Its default values of parameters were used to train the model in the following experiments.
- D-MPNN (Li *et al.* 2022): it adopted 133 features for each atom and 14 features for each bond in a molecule and it performed 6 message-passing iterations on the molecular graph and then used 2 feed-forward layers.
- GAT (Veličković *et al.* 2018): it adopted the same molecular graph features of CMMS-GCL and then used two-layer GAT with 2 heads to a feed-forward neural network. The parameters in the feed-forward neural network are the same as those in CMMS-GCL.
- AttentiveFP (Xiong *et al.* 2020): it implemented the same node features of CMMS-GCL and extra 10-dim bond features as input. Then it used a three-layer message-passing neural network and two-time steps in the message-passing process.

The first five approaches utilized the parameter settings recommended by the original reference literature, while for the latter two methods, we identified the optimal settings.

### 3.3 Performance evaluation on HLM dataset

Table 2 shows the results of 10-fold cross-validation on HLM dataset. Among all the methods, CMMS-GCL achieves the best performance with an average AUC of 0.866±0.013, average Accuracy of 0.805±0.016, average F1 score of 0.853±0.011, and average MCC of 0.570±0.034, which are 1.05%, 1.00%, 0.59%, and 1.42% higher than that of the second-best methods. The results demonstrate the effectiveness of CMMS-GCL in predicting the metabolic stability of compound candidates.

**Table 2.** Performance evaluation of CMMS-GCL with baseline methods on HLM metabolic stability prediction.

Method	AUC	Accuracy	F1 score	MCC
FP-GBDT	0.819±0.019	0.775±0.015	0.831±0.014	0.497±0.031
FP-XGBoost	0.841±0.018	0.789±0.012	0.842±0.010	0.528±0.026
PredMS	0.856±0.015	0.789±0.023	0.848 ± 0.019	0.550±0.101
GCN	0.857 ± 0.017	0.788±0.013	0.835±0.014	0.541±0.036
D-MPNN	0.848±0.013	0.792±0.012	0.841±0.013	0.541±0.030
GAT	0.854±0.019	0.785 ±0.022	0.835±0.017	0.530±0.055
AttentiveFP	0.857 ± 0.013	0.797 ± 0.014	0.840±0.011	0.562 ± 0.033
CMMS-GCL	**0.866** ± **0.013**	**0.805** ± **0.016**	**0.853** ± **0.011**	**0.570** ± **0.034**

The best results are marked in bold and the second best is underlined.

There are two main reasons why our model outperforms baselines. On the one hand, CMMS-GCL combines both compound SMILES sequences with molecular structures, which allows our model to learn more informative representations than baseline methods that use single modality data (i.e. Ryu *et al.* 2022). On the other hand, in the molecular graph structure encoder, we introduce graph contrastive learning to capture the dependencies between local and global structures, which enhances the representation learning of our model. We will demonstrate it in the following ablation study.

### 3.4 Independent evaluation on the external dataset

To further validate the effectiveness and generalizability of our model, we carried out CMMS-GCL and baseline methods on an external dataset. Note that all the methods utilize the HLM dataset for model training and use the external data as a test dataset.

The results in Table 3 show that CMMS-GCL achieved the best performance compared with seven methods across all metrics on 10 times average results. More specifically, CMMS-GCL surpasses the second-best method by 6.60%, 8.17%, 4.45%, and 27.31% in terms of AUC, Accuracy, F1 score, and MCC, respectively. Furthermore, we derived a subset of molecules from the external dataset, which shows low similarity to the HLM dataset. We also compared our CMMS-GCL with various baselines on this subset of molecules with low structural similarity (<0.5) as shown in Table 4. We can observe the improvement of our method upon the second-best by 7.72%, 6.31%, 3.19%, and 38.60% in terms of AUC, Accuracy, F1 score, and MCC, respectively. More importantly, compared with the results on the HLM dataset (Table 2), CMMS-GCL exhibited a more substantial improvement on the external dataset (Tables 3 and 4). In contrast, for baseline methods, especially methods that are based on traditional machine learning, their performance decrease to varying degrees. Therefore, we can make a conclusion that the proposed CMMS-GCL is an effective and robust computational model for predicting the metabolic stability of compounds.

**Table 3.** Performance evaluation of CMMS-GCL with baseline methods on the external datasets.

Method	AUC	Accuracy	F1 score	MCC
FP-GBDT	0.640±0.045	0.710±0.021	0.815±0.016	0.151±0.063
FP-XGBoost	0.659±0.018	0.722±0.017	0.828±0.012	0.141±0.045
PredMS	0.756±0.017	0.754±0.009	0.853 ± 0.004	0.211±0.048
GCN	0.833 ± 0.030	0.771 ± 0.037	0.840 ±0.037	0.443 ± 0.070
D-MPNN	0.760±0.017	0.739±0.010	0.850±0.014	0.220±0.032
GAT	0.819±0.021	0.754±0.050	0.825±0.052	0.411±0.097
AttentiveFP	0.813±0.039	0.748±0.040	0.818±0.042	0.416±0.072
CMMS-GCL	**0.888** ± **0.014**	**0.834** ± **0.026**	**0.891** ± **0.017**	**0.564** ± **0.070**

The best results are marked in bold and the second best is underlined.

**Table 4.** Performance evaluation of CMMS-GCL against baseline methods using a subset of external datasets with low molecular similarity.

Method	AUC	Accuracy	F1 score	MCC
FP-GBDT	0.486±0.059	0.551±0.185	0.739±0.028	0.038±0.062
FP-XGBoost	0.533±0.018	0.610±0.022	0.746±0.019	0.009±0.020
PredMS	0.669±0.017	0.653±0.014	0.783 ± 0.006	0.024±0.033
GCN	0.734±0.036	0.697 ± 0.036	0.775 ±0.037	0.316±0.083
D-MPNN	0.701±0.009	0.638±0.025	0.723±0.012	0.202±0.075
GAT	0.738 ± 0.033	0.690±0.052	0.754±0.068	0.342 ± 0.083
AttentiveFP	0.687±0.026	0.624±0.052	0.664±0.101	0.332±0.061
CMMS-GCL	**0.795** ± **0.026**	**0.741** ± **0.046**	**0.808** ± **0.044**	**0.474** ± **0.056**

The best results are marked in bold and the second best is underlined.

### 3.5 Ablation studies

Our CMMS-GCL consists of an atom similarity-based sequence encoder, a molecular graph structure encoder, an inter-view graph contrastive learning, and a stability predictor, as shown in Fig. 1. In this section, we perform an ablation study on two datasets to evaluate the contributions of the cross-modality encoder and the inter-view graph contrastive learning. Note that in Tables 5 and 6, “w/o CM” denotes the variant of CMMS-GCL only reserves molecular graph structure encoder and stability predictor. “w/o GCL” denotes the variant of CMMS-GCL without inter-view graph contrastive learning. Tables 5 and 6 report the performance comparison between CMMS-GCL and its variants on HLM and the external dataset in terms of four metrics, respectively. It can be discovered that both cross-modality encoder and inter-view graph contrastive learning contribute to the performance improvement of the model. Specifically, in Table 5, compared with the variants w/o CM and w/o GCL, CMMS-GCL improves on AUC by 0.81% and 0.58%, on Accuracy by 1.13% and 0.75%, on F1 score by 1.55% and 0.95%, on MCC by 1.60% and 0.88%, respectively. Moreover, on the external dataset, compared with the variants w/o CM and w/o GCL, CMMS-GCL improves on AUC by 2.19% and 1.49%, on Accuracy by 2.46% and 1.46%, on F1 score by 1.48% and 0.56%, on MCC by 14.40% and 12.13%, respectively.

**Table 5.** Ablation studies of CMMS-GCL, which include two components variants and three augmentation methods variants.

Method	AUC	Accuracy	F1 score	MCC
w/o CM	0.859±0.015	0.796±0.012	0.840±0.010	0.561±0.026
w/o GCL	0.861±0.016	0.799±0.020	0.845±0.021	0.565±0.047
Node Dropping	0.859±0.015	0.798±0.017	0.845±0.012	0.556±0.044
Edge Perturbation	0.863±0.015	0.800±0.023	0.850±0.013	0.557±0.060
Attribute Masking	0.863±0.016	0.802±0.020	0.849±0.013	0.563±0.052
CMMS-GCL	**0.866** ± **0.013**	**0.805** ± **0.016**	**0.853** ± **0.011**	**0.570** ± **0.034**

The best results are marked in bold.

**Table 6.** Ablation studies of CMMS-GCL on external dataset, which include two components variants and three augmentation methods variants.

Method	AUC	Accuracy	F1 score	MCC
w/o CM	0.869±0.012	0.814±0.019	0.878±0.011	0.493±0.085
w/o GCL	0.875±0.011	0.822±0.014	0.886±0.007	0.503±0.055
Node Dropping	0.867±0.015	0.809±0.030	0.876±0.016	0.468±0.118
Edge Perturbation	0.874±0.016	0.806±0.034	0.877±0.017	0.448±0.129
Attribute Masking	0.867±0.014	0.805±0.032	0.876±0.017	0.437±0.118
CMMS-GCL	**0.888** ± **0.014**	**0.834** ± **0.026**	**0.891** ± **0.017**	**0.564** ± **0.070**

The best results are marked in bold.

Moreover, to further validate the superiority of the substructure sampling-based graph augmentation method, we compared it with other three augmentation methods: Node Dropping, Edge Perturbation, and Attribute Masking. The sampling ratios, which determine the fraction of nodes/edges/features selected by these three augmentations, were set to 0.2, 0.4, and 0.4, respectively. The results in Tables 5 and 6 show that the substructure sampling-based method achieves the best performance consistently and improves the second-best method by 0.35%, 0.37%, 0.35%, and 1.24% in terms of AUC, accuracy, F1 score, and MCC, respectively, in Table 5 and improves the second-best method by 1.60%, 3.09%, 1.60%, and 20.51% in terms of AUC, accuracy, F1 score, and MCC, respectively, in Table 6. The results indicate that the substructure sampling-based graph contrastive learning plays a vital role in predicting metabolic stability.

### 3.6 Performance evaluation on novel structural molecules

**Figure 3.**
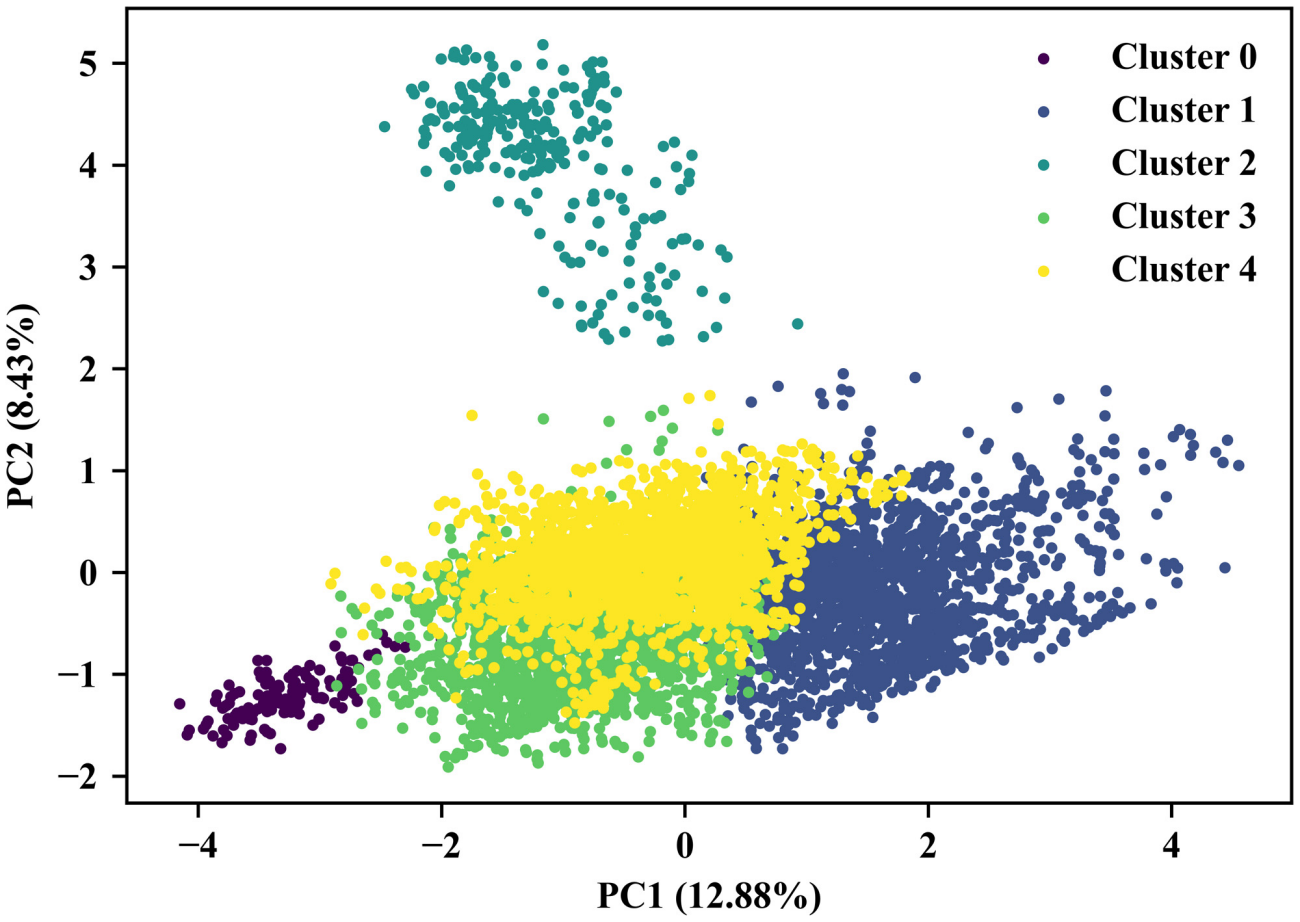
PCA visualization of *K*-means clustering on the HLM dataset, where the values in parentheses correspond to the explained variance ratio.

To validate our CMMS-GCL’s capacity for novel molecular structures, we calculated the Tanimoto similarity among the molecules within the HLM dataset using their ECFP fingerprints. We then performed a *K*-means clustering analysis on the similarity matrix, resulting in five clear clusters. To present our results more tangibly, we employed Principal Component Analysis (PCA) for dimensionality reduction, as depicted in Fig. 3. This dimension reduction allowed us to visualize and interpret the clustering results more effectively, thereby accentuating the efficiency of our CMMS-GCL in identifying distinct molecular structures.

**Figure 4.**
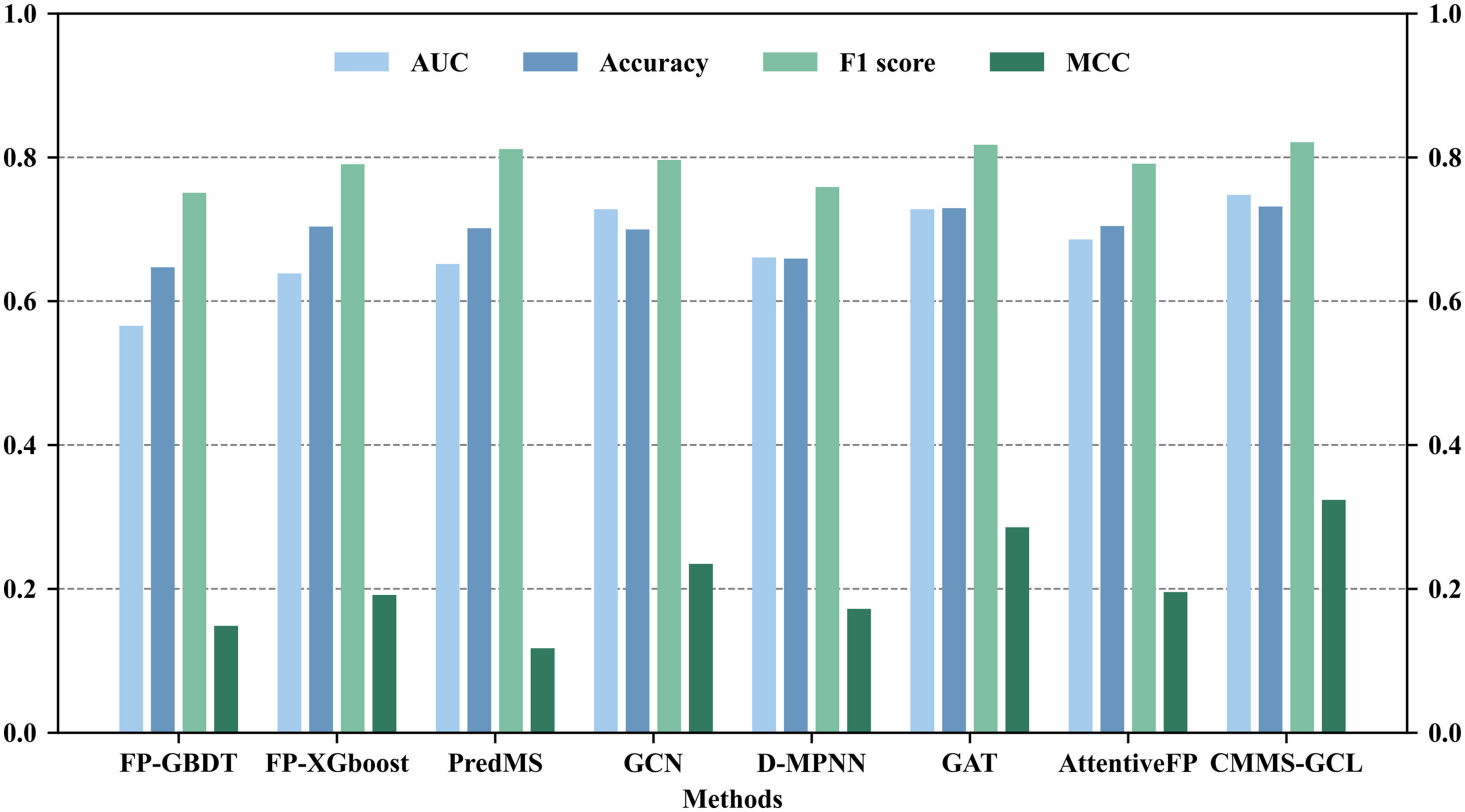
Performance comparison of CMMS-GCL and baseline methods on HLM dataset based on leave-one-cluster-out strategy.

Furthermore, we conducted the leave-one-cluster-out cross-validation on the identified clusters, providing a robust validation of our approach on the molecules with novel structures. We compared our CMMS-GCL against various baseline methods under this leave-one-cluster-out setting in Fig. 4. We can observe that CMMS-GCL outperforms the second-best method by 1.99%, 0.24%, 0.36%, and 3.82% in terms of AUC, Accuracy, F1 score, and MCC, respectively.

In conclusion, the assessment of structurally unique compounds manifests the superior predictive performance of CMMS-GCL, underscoring its competency in handling diverse chemical structures. This revelation accentuates the potential of CMMS-GCL as an invaluable asset for medicinal chemists in pinpointing compounds with novel structures and predicting their respective activities.

### 3.7 Case study on interpretability of CMMS-GCL

Although deep learning is often known as a black-box model, it is crucial to comprehend the prediction mechanism employed by CMMS-GCL for distinguishing stable and unstable molecules, as well as to ascertain its potential influence on lead compound optimization. To thoroughly investigate the interpretability of our model, we embarked on an in-depth examination of its two primary modules, focusing distinctly on the sequence-based and the graph structure-based components.

#### 3.7.1 Sequence-based interpretability

**Figure 5.**
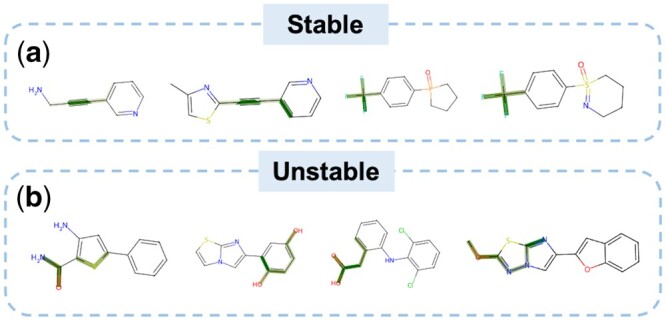
Interpretability case study of crucial functional groups for stable and unstable molecules based on sequences. (a) Four stable molecules with high metabolic stability. (b) Four unstable molecules with low metabolic stability. The atoms and bonds of high/low metabolic stability-specific functional groups are highlighted.

Since the atomic similarity-based sequence encoder module can capture the molecule’s important functional groups through its multihead attention module, we determined that two bonding atoms are regarded as a crucial functional group. The weight of a bond is the average of the weights of its constituent atoms (partial bonds that can be visualized are also averaged together of SMILES symbols) and are highlighted in Fig. 5.

We selected four molecules exhibiting high metabolic stability. The highlighted regions reveal specific areas within the molecules that contribute to metabolic stability or instability. As shown in Fig. 5a, for the first two stable molecules, their highlighted regions exhibit *acetylene* functional groups, which are commonly associated with enhanced stability and are less prone to metabolic reactions (Talele 2020). For the other two molecules, the *trifluoromethyl* group exhibits strong electronegativity and chemical inertness, making it difficult to undergo a transformation during metabolic processes in biological systems (Liang *et al.* 2013).

In contrast, another four molecules display low metabolic stability (i.e. instability). For these unstable molecules prone to metabolism, their prominent functional groups involve various metabolic pathways. As shown in Fig. 5b, the attention scores indicate that specific functional groups, such as *amide*, *thiophene*, *hydroxyl*, *ester*, *methoxy*, and *nitrogen*-containing heterocycles, receive the highest scores, making them crucial for the model’s prediction. Furthermore, *amide* more readily undergo hydrolysis (Casey Laizure *et al.* 2013), and *thiophene* rings can be oxidized by CYP450 enzymes, leading to the formation of *S*-oxides (Gramec *et al.* 2014). Also, *hydroxyl* groups are susceptible to oxidative reactions catalyzed by CYP450 enzymes, such as hydroxylation, resulting in the formation of *quinone* or *quinone*-type phenolic derivatives (Zhang and Tang 2018). *Ester* functional groups are typically susceptible to hydrolysis by esterases in biological systems (Abelian *et al.* 2021), resulting in the formation of corresponding *alcohols* and *acids*. Lastly, the *methoxy* group can potentially be metabolized by enzymes such as cytochrome P450 or *O*-demethylases, converting it to the relevant alcohol and *nitrogen*-containing heterocycles tend to undergo oxidation reactions, forming *N*-oxides on the nitrogen atom, increasing the compound’s polarity, and facilitating its excretion in biological systems(Sharma *et al.* 2018, Makhova *et al.* 2020).

#### 3.7.2 Graph structure-based interpretability

**Figure 6.**
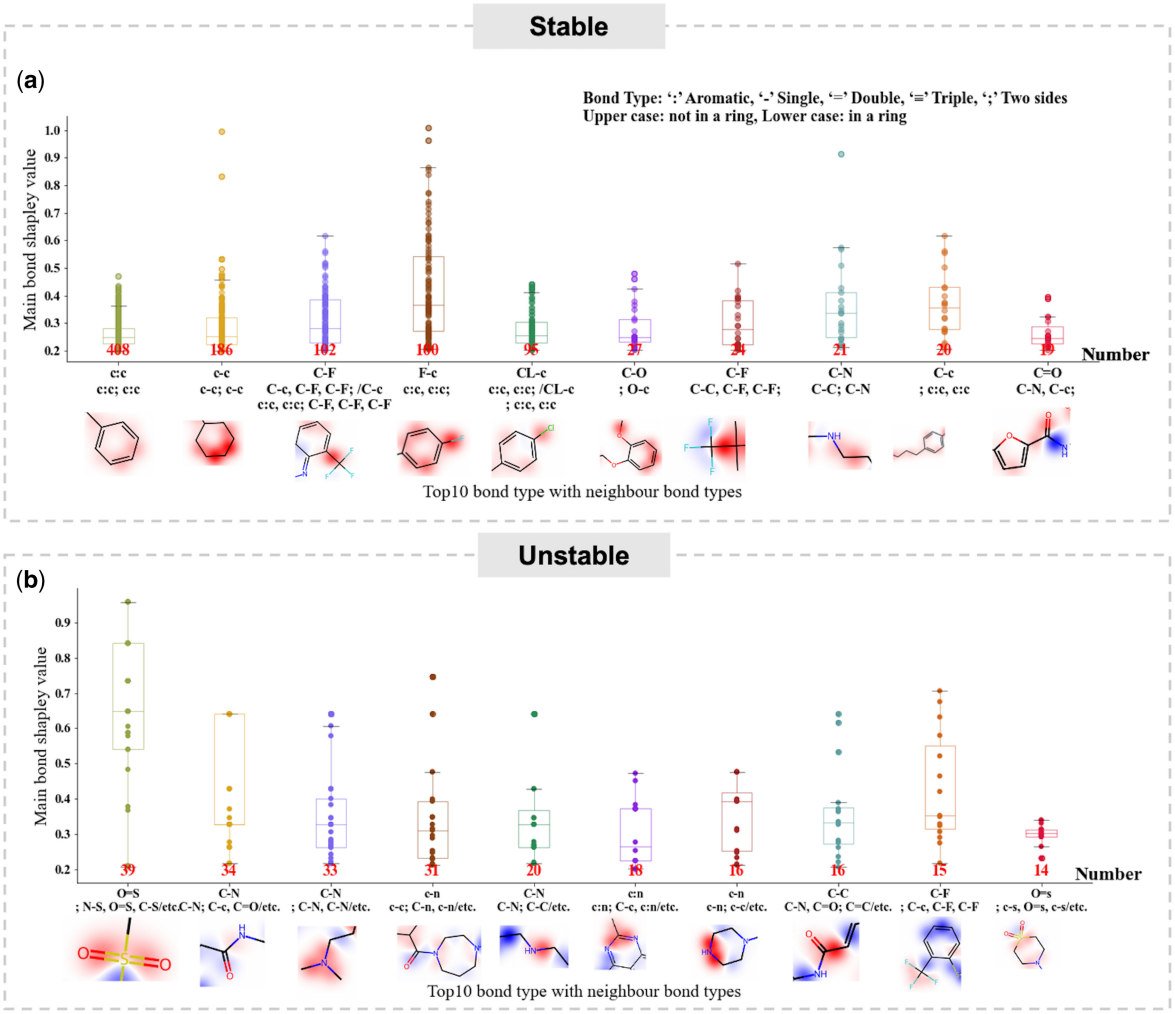
Interpretability case study of crucial functional groups for stable and unstable molecules based on graph structures. (a) The statistical results of the top 10 functional groups with significant impact on stability are arranged in descending order according to their frequency of occurrence. In the substructures, red indicates a positive influence, while blue indicates a negative influence. (b) The statistical results of the top 10 functional groups with significant impact on instability are arranged in descending order of occurrence. In these substructures, red signifies a positive influence, while blue signifies a negative influence.

In terms of the graph structures, we employed the EdgeSHAPer method, which calculates the Shapley value for each edge as if they were individual contributors to the model’s predictive capability (Mastropietro *et al.* 2022). The Shapley value here represents each edge’s (or bond’s) average marginal contribution to the model’s collective prediction of metabolic stability and instability. In order to attain more comprehensive statistics related to the chemical groups that influence metabolic stability, we utilized the trained model on the first fold of the HLM dataset and executed interpretability analysis on the corresponding test set, comprising 383 positive and 202 negative samples. Through a statistical analysis focused on these critical bonds and their neighbour bonds, we ultimately discerned some general statistical patterns. The statistical results of stability and instability are illustrated in Fig. 6. We set a Shapley value threshold of 0.2 and selected the top 10 critical bonds along with their corresponding neighbor bonds based on their occurrence frequency. The substructure bonds were highlighted based on the Shapley value. In Fig. 6a, the top 10 functional groups, which were influenced stability, represented by *phenyl ring*, *ring*, *trifluoromethylbenzene*, *monofluorobenzene*, *chlorobenzene*, *ether*, *trifluoromethyl*, *primary amine*, *alkylbenzene*, and *carbonyl group* have been screened out. They respectively account for approximately 17.9%, 8.2%, 4.5%, 4.4%, 4.2%, 1.2%, 1.1%, 0.9%, 0.9%, and 0.8% of instances where the main bond Shapley value exceeds 0.2. The functional groups that contribute strongly to stability are primarily the top five, accounting for 39.2% of instances, and *trifluoromethylbenzene* generally has a high Shapley value. Our results are essentially consistent with the literature (Wojtuch *et al.* 2021) and sequence-based interpretability.

In Fig. 6b, the top 10 functional groups most prone to metabolic instability are *sulfonate*, *amide*, *secondary amine*, *diazaheterocycle*, *primary amine*, *diazabenzene*, *amide* or *carboxylic acid*, *trifluorobenzene*, and *sulfonate*, with respective proportions of 6.6%, 5.8%, 5.6%, 5.2%, 3.4%, 3.0%, 2.7%, 2.7%, 2.5%, and 2.4% of instances where the main bond Shapley value exceeds 0.2. Due to the extensive variety of metabolic pathway types (Du *et al.* 2022), there are many functional groups influencing metabolic instability. Consequently, the distribution of these functional groups tends to be quite dispersed. Our results are essentially consistent with the literature (Wojtuch *et al.* 2021) and sequence-based interpretability, with the exception of *trifluorobenzene*. The occurrence probability of *trifluorobenzene* in metabolic instability is significantly lower than its occurrence in the cases of stability. This discrepancy may potentially be attributed to statistical errors.

In summary, the alignment of our findings from the model and existing literature demonstrates the interpretability of CMMS-GCL, highlighting functional groups significantly associated with metabolic stability. This understanding enables the elucidation of the reasons behind a molecule’s specific stability or instability properties. Meanwhile, the insights derived from our analysis can contribute to the guidance of molecular design and the optimization of lead compounds.

## 4 Discussion and conclusion

Assessing metabolic stability is essential for drug discovery and development. However, the high cost and risk associated with wet-lab experiments make it challenging to conduct this assessment. Therefore, computational methods can be a fast and effective complementary approach for predicting metabolic stability. However, it is noted that few computational methods have been developed for such tasks.

In this article, we present CMMS-GCL, a novel cross-modality graph contrastive learning model named CMMS-GCL for predicting the metabolic stability of compound candidates. In the model, we design a dual-channel strategy to learn representations for molecules from two different modalities, i.e. SMILES sequence and graph structure. Especially, with the SMILES sequence data as inputs, a multihead BiGRU-based encoder is designed to learn the sequence representations of molecules by fully preserving the local chemical context of atoms. The introduction of multihead BiGRU enables the identification of crucial functional groups specific to metabolic stability. Meanwhile, we propose a GIN-based encoder to learn structure representations from the molecule structure, where graph contrastive learning is further introduced to enhance representation learning by capturing dependencies between local and global structures. Comprehensive experimental results on two datasets demonstrate that our proposed CMMS-GCL outperforms seven state-of-the-art methods in predicting metabolic stability. Additionally, we performed case studies on various stable and unstable molecules, demonstrating the interpretability of key functional groups recognized by CMMS-GCL in sequence analysis. Moreover, we conducted a statistical interpretability analysis utilizing the graph structure, further emphasizing the advantages and potential utility of our method. These statistical analyses not only corroborate our model but also provide valuable guidance for future candidate drug screening and lead compound optimization.

Although the CMMS-GCL method shows promising results in predicting metabolic stability, it still faces several limitations, including the challenges in effectively incorporating unlabeled data with experimental data and the discrepancies between *in vitro* data and *in vivo* predictions due to factors such as complex *in vivo* metabolism, physiological factors, and interindividual variability. To address these challenges, semisupervised and self-supervised learning techniques can be designed to integrate unlabeled data with the existing labeled data, thereby enhancing learning accuracy and overall prediction performance. Moreover, the incorporation of diverse data sources, validation of model performance using *in vivo* data, and exploration of methodologies to account for physiological factors and interindividual variability can also be investigated in future research.
